# Supplementary material for: Effectiveness and safety of continuous low-molecular-weight heparin versus switching to direct oral anticoagulants in cancer-associated venous thrombosis
Source: Nat Commun. 2024 Jul 5;15:5657. doi: 10.1038/s41467-024-50037-1 (PMC11229502; doi:10.1038/s41467-024-50037-1)
Supplement: Supplementary file 1 — Supplementary Information [file 41467_2024_50037_MOESM1_ESM.docx]

# Supplementary Figure 1. Case validation for active cancer, hospitalization due to VTE, and major bleeding

**
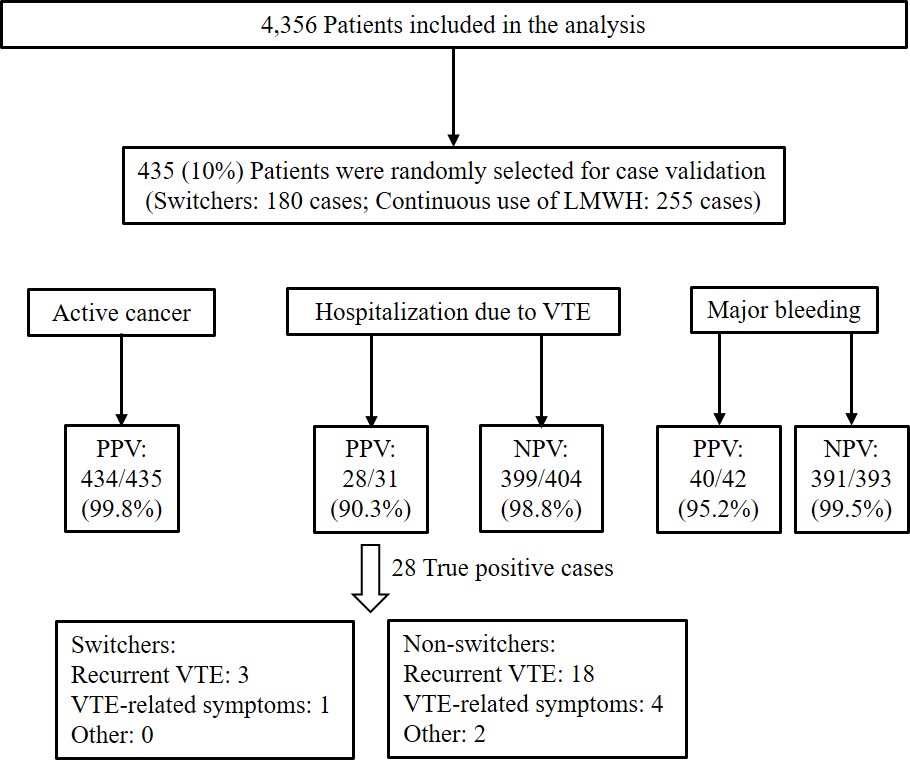
**

Electronic medical records of active cancer, hospitalization due to venous thromboembolism (VTE), and major bleeding acquired from the Clinical Data Analysis and Reporting System (CDARS) were validated against clinical notes from the Clinical Management System for a 10% randomly selected patient population in the study cohort. Positive predictive values (PPVs) and negative predictive values (NPVs) were calculated for data validation. Switchers were defined as patients who switched to DOACs (apixaban, dabigatran, edoxaban, or rivaroxaban) after receiving LMWH (enoxaparin, tinzaparin, or nadroparin) treatment for any duration. Non-switchers were defined as patients who consistently received LMWH.

# Supplementary Figure 2. Patient selection flowchart in landmark analysis


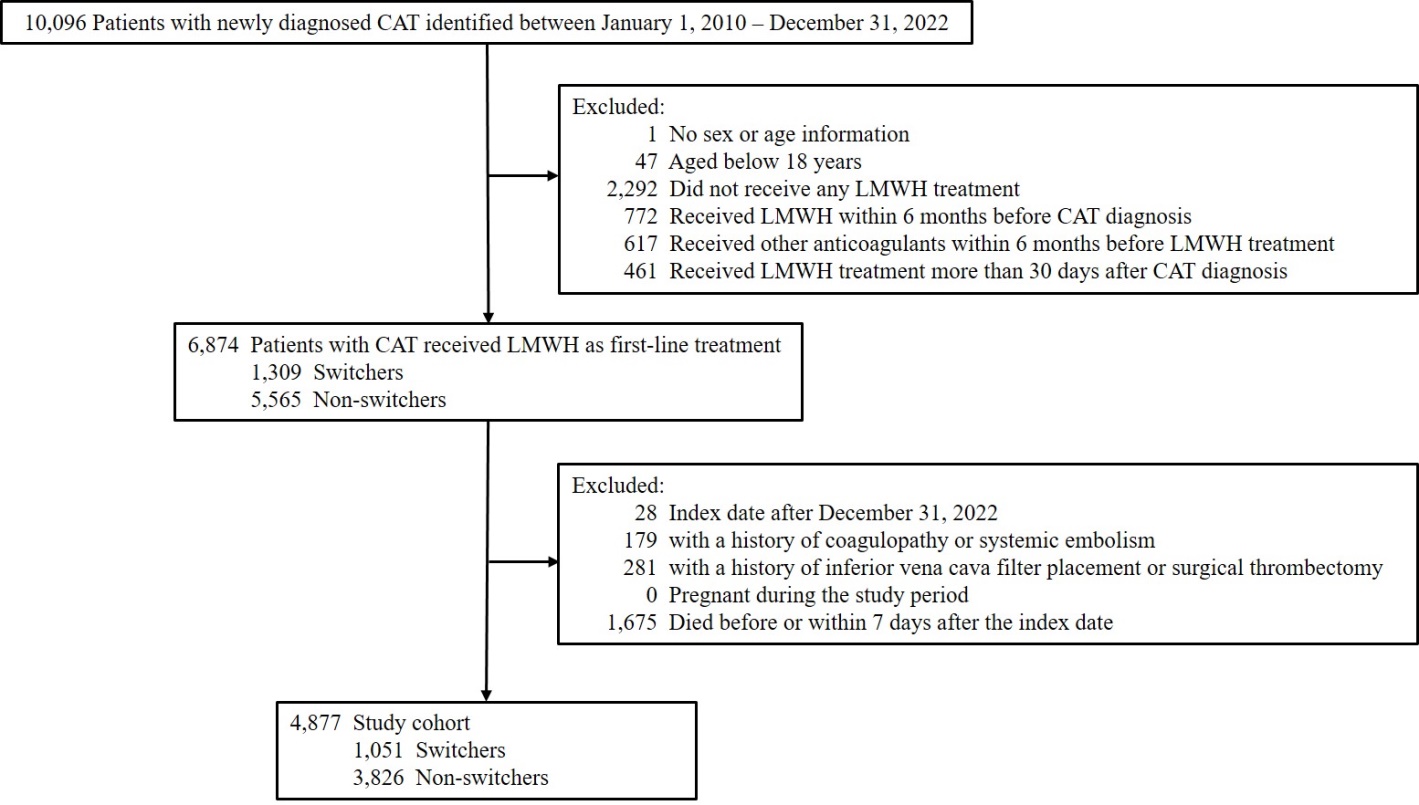


Cohort selection for landmark analysis for the effectiveness and safety of continuous low-molecular-weight heparin (LMWH) versus switching to direct oral anticoagulants (DOACs) among patients with cancer-associated venous thrombosis (CAT). Switchers were defined as patients who switched to DOACs after receiving LMWH treatment during the initial 30 days following the incident of CAT. Non-switchers were defined as patients who consistently received LMWH during the initial 30 days following the incident of CAT.

# Supplementary Figure 3. Landmark analysis design


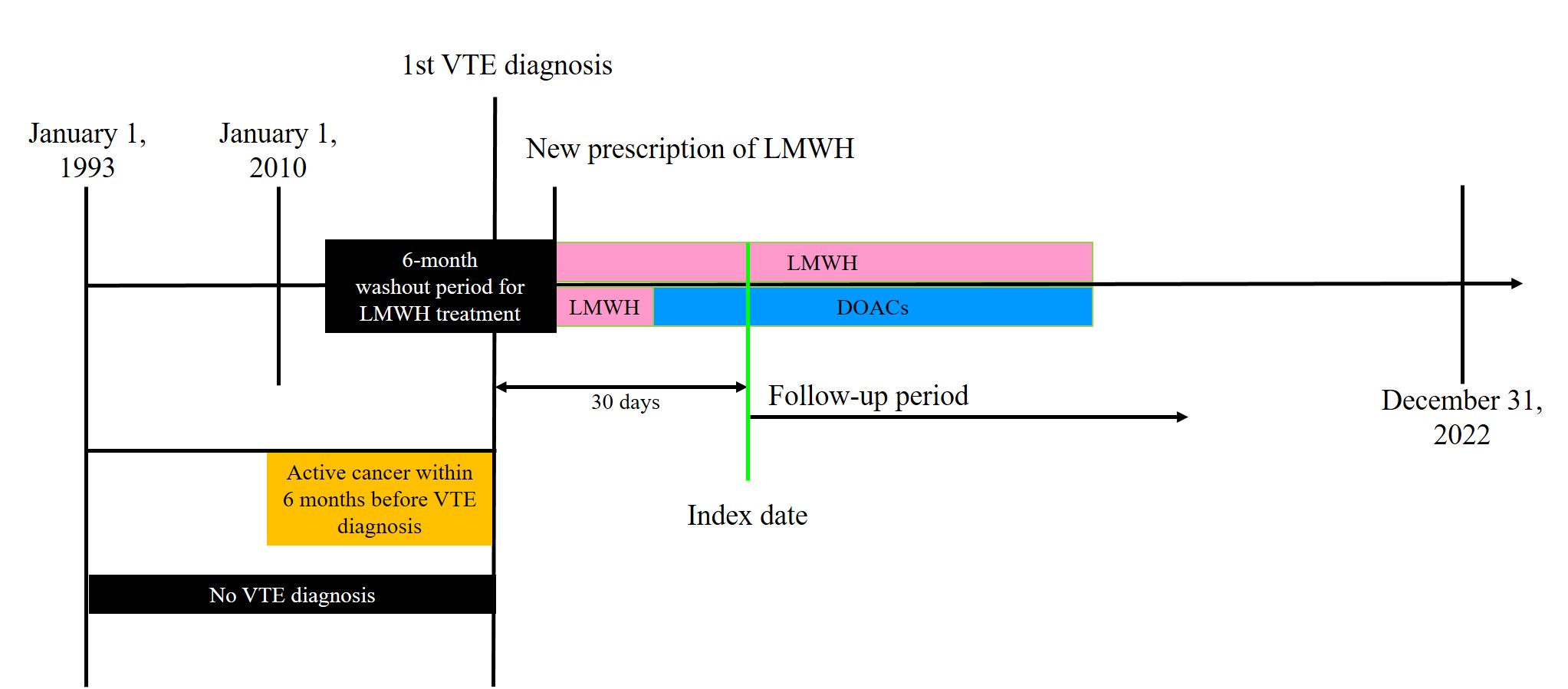


Patients with cancer-associated venous thrombosis (CAT) were identified as those with active cancer followed by an incident diagnosis of venous thromboembolism (VTE) between January 1, 2010, and December 31, 2022. Active cancer (yellow band) was defined as a new cancer diagnosis, recurrent cancer diagnosis, presence of metastasis, any cancer-related treatment, or palliative care within the six months prior to the first VTE diagnosis. The start of initial LMWH treatment was determined as the date of the first recorded LMWH prescription following the CAT diagnosis. Patients with a history of LMWH treatment within six months prior to the initial LMWH treatment were excluded as LMWH-experienced. In this landmark analysis, the study cohort was divided into two groups based on whether they switched to direct oral anticoagulant (DOAC, blue band) treatment from low-molecular-weight heparin (LMWH, pink band) during the initial 30 days following the incident of CAT or not. The index date (green line) in both groups was the 30^th^ day after the incident CAT. Patients were followed up from the index date for six months or until the occurrence of the outcome, death, the end of anticoagulation treatment, or the end of the study period (December 31, 2022), whichever occurred first.

# Supplementary Table 1. Sensitivity analysis: Extended the follow-up period to one year

| Outcomes | Events/Incidence  (per 100 person-year) | | Unweighted | | Weighted | | Competing risk | |
| --- | --- | --- | --- | --- | --- | --- | --- | --- |
|  | Non-switchers  (N=2,656) | Switchers (N=1,700) | Hazard Ratio  (95% CI) | P value | Hazard Ratio  (95% CI) | P value | Subdistribution  Hazard Ratio  (95% CI) | P value |
| Hospitalization due to VTE | 166/15 | 64/7 | 0.50 (0.38-0.67) | 2.79e-6 | 0.51 (0.38-0.69) | 1.13e-5 | 0.62 (0.47-0.83) | 0.001 |
| Hospitalization due to DVT | 112/10 | 37/4 | 0.43 (0.30-0.62) | 8.86e-6 | 0.44 (0.30-0.65) | 3.07e-5 | 0.57 (0.39-0.82) | 0.002 |
| Hospitalization due to PE | 58/5 | 29/3 | 0.67 (0.43-1.04) | 0.074 | 0.67 (0.42-1.07) | 0.092 | 0.77 (0.48-1.23) | 0.270 |
| Major bleeding | 226/20 | 163/19 | 0.98 (0.80-1.20) | 0.852 | 1.01 (0.82-1.24) | 0.932 | 1.17 (0.95-1.44) | 0.130 |
| ICH | 28/02 | 13/1 | 0.62 (0.32-1.20) | 0.158 | 0.64 (0.32-1.25) | 0.193 | 0.73 (0.37-1.45) | 0.370 |
| GI bleeding | 56/5 | 49/5 | 1.17 (0.80-1.72) | 0.418 | 1.21 (0.81-1.79) | 0.352 | 1.45 (0.98-2.15) | 0.063 |
| Bleeding of other critical sites | 145/13 | 107/12 | 1.00 (0.78-1.29) | 0.971 | 1.04 (0.80-1.35) | 0.779 | 1.20 (0.93-1.56) | 0.160 |
| All-cause mortality | 1937/164 | 905/97 | 0.62 (0.58-0.67) | 5.65e-32 | 0.68 (0.62-0.73) | 8.41e-21 | - | - |

N: Number of patients; CI: Confidence interval; VTE: Venous thromboembolism; DVT: deep vein thrombosis; PE: pulmonary embolism; ICH: intracranial hemorrhage; GI bleeding: gastrointestinal bleeding. All P values were from 2-sided tests and results were deemed statistically significant at P < 0.05.

# Supplementary Table 2. Sensitivity analysis: Excluded patients with basal cell carcinoma, squamous-cell skin carcinoma, lymphoma, acute leukemia or myeloproliferative neoplasm before or during the study period

| Outcomes | Events/Incidence  (per 100 person-year) | | Unweighted | | Weighted | | Competing risk | |
| --- | --- | --- | --- | --- | --- | --- | --- | --- |
|  | Non-switchers  (N=2,503) | Switchers (N=1,597) | Hazard Ratio  (95% CI) | P value | Hazard Ratio  (95% CI) | P value | Subdistribution  Hazard Ratio  (95% CI) | P value |
| Hospitalization due to VTE | 132/20 | 51/10 | 0.52 (0.37-0.71) | 5.85e-5 | 0.52 (0.37-0.72) | 1.14e-4 | 0.62 (0.45-0.86) | 0.004 |
| Hospitalization due to DVT | 87/13 | 26/5 | 0.40 (0.26-0.62) | 4.31e-5 | 0.41 (0.26-0.65) | 1.21e-4 | 0.52 (0.34-0.80) | 0.003 |
| Hospitalization due to PE | 48/7 | 26/5 | 0.73 (0.45-1.18) | 0.202 | 0.71 (0.43-1.16) | 0.171 | 0.80 (0.48-1.33) | 0.390 |
| Major bleeding | 189/28 | 132/26 | 0.97 (0.78-1.22) | 0.819 | 1.00 (0.80-1.26) | 0.979 | 1.13 (0.90-1.43) | 0.280 |
| ICH | 24/3 | 11/2 | 0.63 (0.31-1.29) | 0.206 | 0.64 (0.30-1.34) | 0.233 | 0.72 (0.34-1.51) | 0.380 |
| GI bleeding | 48/7 | 36/7 | 1.03 (0.67-1.58) | 0.905 | 1.07 (0.69-1.68) | 0.752 | 1.25 (0.80-1.95) | 0.330 |
| Bleeding of other critical sites | 119/17 | 87/17 | 1.03 (0.78-1.36) | 0.843 | 1.05 (0.79-1.41) | 0.721 | 1.18 (0.88-1.57) | 0.260 |
| All-cause mortality | 1560/222 | 700/133 | 0.62 (0.56-0.67) | 1.83e-6 | 0.67 (0.61-0.74) | 6.77e-17 | - | - |

N: Number of patients; CI: Confidence interval; VTE: Venous thromboembolism; DVT: deep vein thrombosis; PE: pulmonary embolism; ICH: intracranial hemorrhage; GI bleeding: gastrointestinal bleeding. All P values were from 2-sided tests and results were deemed statistically significant at P < 0.05.

# Supplementary Table 3. Sensitivity analysis: Excluded patients with a short follow-up period (index date later than July 4, 2022)

| Outcomes | Events/Incidence  (per 100 person-year) | | Unweighted | | Weighted | | Competing risk | |
| --- | --- | --- | --- | --- | --- | --- | --- | --- |
|  | Non-switchers  (N=2,626) | Switchers (N=1,525) | Hazard Ratio  (95% CI) | P value | Hazard Ratio  (95% CI) | P value | Subdistribution  Hazard Ratio  (95% CI) | P value |
| Hospitalization due to VTE | 148/21 | 51/10 | 0.49 (0.35-0.67) | 9.99e-6 | 0.49 (0.35-0.68) | 1.56e-5 | 0.59 (0.43-0.82) | 0.001 |
| Hospitalization due to DVT | 99/14 | 27/5 | 0.39 (0.25-0.59) | 1.32e-5 | 0.40 (0.26-0.63) | 4.97e-5 | 0.50 (0.33-0.77) | 0.001 |
| Hospitalization due to PE | 52/7 | 25/5 | 0.69 (0.43-1.12) | 0.132 | 0.65 (0.40-1.06) | 0.085 | 0.76 (0.46-1.26) | 0.290 |
| Major bleeding | 192/27 | 124/24 | 0.96 (0.77-1.20) | 0.720 | 0.99 (0.79-1.26) | 0.961 | 1.14 (0.90-1.44) | 0.270 |
| ICH | 24/3 | 10/2 | 0.61 (0.29-1.27) | 0.188 | 0.60 (0.28-1.29) | 0.192 | 0.70 (0.33-1.49) | 0.360 |
| GI bleeding | 48/6 | 36/7 | 1.09 (0.71-1.68) | 0.697 | 1.16 (0.74-1.80) | 0.522 | 1.34 (0.87-2.08) | 0.190 |
| Bleeding of other critical sites | 122/17 | 80/15 | 0.98 (0.74-1.31) | 0.914 | 1.02 (0.76-1.37) | 0.904 | 1.16 (0.86-1.55) | 0.330 |
| All-cause mortality | 1612/215 | 665/125 | 0.60 (0.55-0.66) | 4.44e-28 | 0.67 (0.61-0.73) | 2.02e-17 | - | - |

N: Number of patients; CI: Confidence interval; VTE: Venous thromboembolism; DVT: deep vein thrombosis; PE: pulmonary embolism; ICH: intracranial hemorrhage; GI bleeding: gastrointestinal bleeding. All P values were from 2-sided tests and results were deemed statistically significant at P < 0.05.

# Supplementary Table 4. Sensitivity analysis: Excluded patients diagnosed with cancer-associated venous thrombosis on or after January 1, 2020

| Outcomes | Events/Incidence  (per 100 person-year) | | Unweighted | | Weighted | | Competing risk | |
| --- | --- | --- | --- | --- | --- | --- | --- | --- |
|  | Non-switchers  (N=2,347) | Switchers  (N=796) | Hazard Ratio  (95% CI) | P value | Hazard Ratio  (95% CI) | P value | Subdistribution  Hazard Ratio  (95% CI) | P value |
| Hospitalization due to VTE | 137/21 | 29/10 | 0.49 (0.33-0.74) | 5.75e-4 | 0.44 (0.29-0.67) | 1.26e-4 | 0.60 (0.40-0.89) | 0.012 |
| Hospitalization due to DVT | 93/14 | 15/5 | 0.38 (0.22-0.65) | 4.93e-4 | 0.34 (0.19-0.60) | 1.78e-4 | 0.49 (0.28-0.84) | 0.009 |
| Hospitalization due to PE | 47/7 | 14/5 | 0.71 (0.39-1.29) | 0.256 | 0.62 (0.33-1.16) | 0.133 | 0.72 (0.38-1.36) | 0.310 |
| Major bleeding | 170/26 | 61/22 | 0.88 (0.66-1.19) | 0.410 | 0.96 (0.70-1.32) | 0.800 | 1.15 (0.85-1.56) | 0.360 |
| ICH | 21/3 | 4/1 | 0.46 (0.16-1.35) | 0.159 | 0.47 (0.15-1.42) | 0.178 | 0.58 (0.19-1.78) | 0.340 |
| GI bleeding | 38/6 | 18/6 | 1.14 (0.65-1.99) | 0.652 | 1.24 (0.67-2.30) | 0.490 | 1.44 (0.81-2.57) | 0.220 |
| Bleeding of other critical sites | 113/17 | 40/14 | 0.88 (0.61-1.26) | 0.478 | 0.95 (0.65-1.41) | 0.813 | 1.16 (0.80-1.69) | 0.440 |
| All-cause mortality | 1411/207 | 306/104 | 0.52 (0.46-0.59) | 1.39e-24 | 0.64 (0.56-0.74) | 1.70e-10 | - | - |

N: Number of patients; CI: Confidence interval; VTE: Venous thromboembolism; DVT: deep vein thrombosis; PE: pulmonary embolism; ICH: intracranial hemorrhage; GI bleeding: gastrointestinal bleeding. All P values were from 2-sided tests and results were deemed statistically significant at P < 0.05.

# Supplementary Table 5. Sensitivity analysis: The index year was included as a confounder

| Outcomes | Events/Incidence  (per 100 person-year) | | Unweighted | | Weighted | | Competing risk | |
| --- | --- | --- | --- | --- | --- | --- | --- | --- |
|  | Non-switchers  (N=2,656) | Switchers (N=1,700) | Hazard Ratio  (95% CI) | P value | Hazard Ratio  (95% CI) | P value | Subdistribution  Hazard Ratio  (95% CI) | P value |
| Hospitalization due to VTE | 148/21 | 53/10 | 0.48 (0.35-0.65) | 3.73e-6 | 0.50 (0.31-0.80) | 0.004 | 0.78 (0.53-1.14) | 0.200 |
| Hospitalization due to DVT | 99/14 | 28/5 | 0.38 (0.25-0.58) | 5.73e-6 | 0.36 (0.21-0.61) | 1.35e-4 | 0.75 (0.46-1.22) | 0.250 |
| Hospitalization due to PE | 52/7 | 26/5 | 0.68 (0.42-1.08) | 0.104 | 0.77 (0.36-1.61) | 0.481 | 0.80 (0.44-1.45) | 0.460 |
| Major bleeding | 192/27 | 139/26 | 1.01 (0.81-1.26) | 0.931 | 0.96 (0.71-1.28) | 0.770 | 1.14 (0.87-1.49) | 0.350 |
| ICH | 24/3 | 11/2 | 0.63 (0.31-1.29) | 0.205 | 0.50 (0.21-1.16) | 0.105 | 0.57 (0.25-1.30) | 0.180 |
| GI bleeding | 48/6 | 41/7 | 1.17 (0.77-1.78) | 0.458 | 0.90 (0.55-1.47) | 0.669 | 1.12 (0.69-1.81) | 0.650 |
| Bleeding of other critical sites | 122/17 | 89/16 | 1.02 (0.78-1.35) | 0.865 | 1.08 (0.75-1.57) | 0.674 | 1.31 (0.92-1.85) | 0.130 |
| All-cause mortality | 1629/216 | 729/129 | 0.61 (0.56-0.67) | 6.53e-28 | 0.58 (0.50-0.67) | 9.92e-14 | - | - |

N: Number of patients; CI: Confidence interval; VTE: Venous thromboembolism; DVT: deep vein thrombosis; PE: pulmonary embolism; ICH: intracranial hemorrhage; GI bleeding: gastrointestinal bleeding. All P values were from 2-sided tests and results were deemed statistically significant at P < 0.05.

# Supplementary Table 6. Sensitivity analysis: The end date of anticoagulation treatment was added as a follow-up endpoint

| Outcomes | Events/Incidence  (per 100 person-year) | | Unweighted | | Weighted | | Competing risk | |
| --- | --- | --- | --- | --- | --- | --- | --- | --- |
|  | Non-switchers  (N=2,656) | Switchers (N=1,700) | Hazard Ratio  (95% CI) | P value | Hazard Ratio  (95% CI) | P value | Subdistribution  Hazard Ratio  (95% CI) | P value |
| Hospitalization due to VTE | 69/19 | 40/10 | 0.53 (0.36-0.79) | 0.002 | 0.54 (0.36-0.81) | 0.003 | 0.61 (0.41-0.91) | 0.015 |
| Hospitalization due to DVT | 43/12 | 23/6 | 0.49 (0.30-0.82) | 0.006 | 0.50 (0.30-0.84) | 0.009 | 0.59 (0.35-0.97) | 0.037 |
| Hospitalization due to PE | 28/8 | 18/4 | 0.60 (0.33-1.08) | 0.087 | 0.61 (0.33-1.11) | 0.103 | 0.67 (0.35-1.26) | 0.210 |
| Major bleeding | 90/25 | 96/25 | 1.06 (0.79-1.41) | 0.695 | 1.10 (0.82-1.49) | 0.528 | 1.18 (0.88-1.59) | 0.260 |
| ICH | 5/1 | 7/2 | 1.36 (0.43-4.30) | 0.604 | 1.67 (0.51-5.45) | 0.393 | 1.85 (0.53-6.44) | 0.330 |
| GI bleeding | 21/6 | 25/6 | 1.14 (0.63-2.03) | 0.668 | 1.14 (0.63-2.06) | 0.662 | 1.30 (0.74-2.27) | 0.360 |
| Bleeding of other critical sites | 64/18 | 66/17 | 1.04 (0.74-1.47) | 0.828 | 1.08 (0.75-1.55) | 0.675 | 1.13 (0.78-1.63) | 0.520 |
| All-cause mortality | 642/174 | 332/83 | 0.49 (0.43-0.56) | 2.34e-25 | 0.54 (0.47-0.62) | 2.51e-18 | - | - |

N: Number of patients; CI: Confidence interval; VTE: Venous thromboembolism; DVT: deep vein thrombosis; PE: pulmonary embolism; ICH: intracranial hemorrhage; GI bleeding: gastrointestinal bleeding. All P values were from 2-sided tests and results were deemed statistically significant at P < 0.05.

# Supplementary Table 7. Sensitivity analysis: The landmark analysis

| Outcomes | Events/Incidence  (per 100 person-year) | | Unweighted | | Weighted | | Competing risk | |
| --- | --- | --- | --- | --- | --- | --- | --- | --- |
|  | Non-switchers  (N=3,826) | Switchers (N=1,051) | Hazard Ratio  (95% CI) | P value | Hazard Ratio  (95% CI) | P value | Subdistribution  Hazard Ratio  (95% CI) | P value |
| Hospitalization due to VTE | 92/30 | 25/13 | 0.57 (0.37-0.89) | 0.013 | 0.54 (0.33-0.89) | 0.015 | 0.60 (0.39-0.94) | 0.027 |
| Hospitalization due to DVT | 59/19 | 12/6 | 0.43 (0.23-0.79) | 0.007 | 0.35 (0.18-0.68) | 0.002 | 0.43 (0.23-0.80) | 0.008 |
| Hospitalization due to PE | 35/11 | 14/7 | 0.85 (0.46-1.58) | 0.604 | 0.90 (0.45-1.79) | 0.770 | 0.93 (0.48-1.81) | 0.830 |
| Major bleeding | 93/30 | 48/26 | 1.07 (0.76-1.52) | 0.701 | 0.98 (0.68-1.41) | 0.919 | 1.08 (0.75-1.56) | 0.660 |
| ICH | 6/2 | 1/1 | 0.35 (0.04-2.87) | 0.325 | 0.31 (0.04-2.62) | 0.283 | 0.33 (0.02-4.34) | 0.400 |
| GI bleeding | 22/7 | 14/7 | 1.31 (0.67-2.56) | 0.432 | 1.27 (0.64-2.52) | 0.490 | 1.53 (0.74-3.14) | 0.250 |
| Bleeding of other critical sites | 65/21 | 34/18 | 1.09 (0.72-1.65) | 0.694 | 0.96 (0.62-1.49) | 0.871 | 1.05 (0.68-1.61) | 0.840 |
| All-cause mortality | 579/181 | 170/88 | 0.61 (0.51-0.72) | 1.09e-8 | 0.61 (0.51-0.73) | 1.18e-7 | - | - |

N: Number of patients; CI: Confidence interval; VTE: Venous thromboembolism; DVT: deep vein thrombosis; PE: pulmonary embolism; ICH: intracranial hemorrhage; GI bleeding: gastrointestinal bleeding. All P values were from 2-sided tests and results were deemed statistically significant at P < 0.05.

# Supplementary Table 8. Sensitivity analysis: The multiple imputation analysis

| Outcomes | Events/Incidence  (per 100 person-year) | | Hazard Ratio (95% CI) | P value |
| --- | --- | --- | --- | --- |
|  | Non-Switchers  (N=2,656) | Switchers (N=1,700) |  |  |
| Hospitalization due to VTE | 148/21 | 53/10 | 0.45 (0.29-0.70) | 5.89e-4 |
| Hospitalization due to DVT | 99/14 | 28/5 | 0.43 (0.24-0.77) | 0.005 |
| Hospitalization due to PE | 52/7 | 26/5 | 0.50 (0.24-1.01) | 0.054 |
| Major bleeding | 192/27 | 139/26 | 1.02 (0.74-1.42) | 0.886 |
| ICH | 24/3 | 11/2 | 1.59 (0.35-7.15) | 0.537 |
| GI bleeding | 48/6 | 41/7 | 1.07 (0.58-1.95) | 0.828 |
| Bleeding of other critical sites | 122/17 | 89/16 | 0.99 (0.66-1.48) | 0.947 |
| All-cause mortality | 1629/216 | 729/129 | 0.51 (0.44-0.59) | 6.66e-16 |

N: Number of patients; CI: Confidence interval; VTE: Venous thromboembolism; DVT: deep vein thrombosis; PE: pulmonary embolism; ICH: intracranial hemorrhage; GI bleeding: gastrointestinal bleeding. All P values were from 2-sided tests and results were deemed statistically significant at P < 0.05.

# Supplementary Table 9. Sensitivity analysis: Excluded patients whose anticoagulation treatment duration <30 days or ≥180 days

| Outcomes | Events/Incidence  (per 100 person-year) | | Unweighted | | Weighted | | Competing risk | |
| --- | --- | --- | --- | --- | --- | --- | --- | --- |
|  | Non-switchers  (N=1,010) | Switchers  (N=763) | Hazard Ratio  (95% CI) | P value | Hazard Ratio  (95% CI) | P value | Subdistribution  Hazard Ratio  (95% CI) | P value |
| Hospitalization due to VTE | 74/27 | 29/14 | 0.52 (0.34-0.79) | 0.002 | 0.53 (0.34-0.82) | 0.004 | 0.56 (0.36-0.86) | 0.008 |
| Hospitalization due to DVT | 45/16 | 13/6 | 0.38 (0.21-0.71) | 0.002 | 0.39 (0.21-0.73) | 0.003 | 0.41 (0.22-0.78) | 0.007 |
| Hospitalization due to PE | 30/11 | 17/8 | 0.77 (0.42-1.39) | 0.381 | 0.76 (0.41-1.40) | 0.379 | 0.78 (0.41-1.47) | 0.440 |
| Major bleeding | 87/32 | 72/35 | 1.12 (0.82-1.54) | 0.461 | 1.05 (0.76-1.45) | 0.751 | 1.07 (0.78-1.48) | 0.670 |
| ICH | 11/4 | 3/1 | 0.37 (0.10-1.31) | 0.122 | 0.35 (0.10-1.30) | 0.117 | 0.41 (0.10-1.74) | 0.230 |
| GI bleeding | 20/7 | 23/11 | 1.56 (0.86-2.85) | 0.144 | 1.55 (0.84-2.86) | 0.162 | 1.51 (0.78-2.94) | 0.220 |
| Bleeding of other critical sites | 56/20 | 47/23 | 1.14 (0.77-1.67) | 0.520 | 1.02 (0.68-1.52) | 0.931 | 1.00 (0.66-1.51) | 0.990 |
| All-cause mortality | 710/248 | 445/208 | 0.84 (0.75-0.95) | 0.005 | 0.84 (0.74-0.95) | 0.006 | - | - |

N: Number of patients; CI: Confidence interval; VTE: Venous thromboembolism; DVT: deep vein thrombosis; PE: pulmonary embolism; ICH: intracranial hemorrhage; GI bleeding: gastrointestinal bleeding. All P values were from 2-sided tests and results were deemed statistically significant at P < 0.05.

# Supplementary Table 10. List of diagnoses and procedure codes in this study

| Comorbidities | ICD-9-CM diagnosis codes | ICD-9-CM procedure codes (details) |
| --- | --- | --- |
| All malignancies | 140-209 |  |
| Lip, oral cavity and pharynx | 140-149 |  |
| Digestive organs | 150-159 |  |
| Liver | 155 |  |
| Respiratory system | 160-165 |  |
| Bone skin and soft tissue | 170-173, 176 |  |
| Breast and genital organs | 158, 174-175, 179-187 |  |
| Urinary organs | 188-189 |  |
| Eye, brain and other central nervous system endocrine glands | 190-194 |  |
| Lymphatic and hematopoietic tissue | 200-209 |  |
| Metastasis | 196-199 |  |
| Obesity | 278.0, V85.3-4 |  |
| Tobacco use disorder | 305.1, V15.82 |  |
| Alcohol use disorder | 291, 303, 305.0, 357.5, 425.5, 535.3, 571.0-571.3, 790.3, 980, V11.3 |  |
| Drug abuse | 292, 304, 305.2-9 |  |
| Coagulopathy | 286, 287.1, 287.3-287.5 |  |
| Systemic embolism | 444-445 |  |
| Pregnancy | 630-679 |  |
| Hypertension | 401-405, 437.2 |  |
| Hyperlipidemia | 272.0-272.4 |  |
| Atrial fibrillation | 427.3 |  |
| Congestive heart failure (CHF) | 398.91, 402.01, 402.11, 402.91, 404.01, 404.03, 404.11, 404.13, 404.91, 404.93, 428 |  |
| Ischemic stroke | 433, 434, 436-438 |  |
| Vascular disease | 410-414, 443.8, 443.9 |  |
| Venous Thromboembolism (VTE) |  |  |
| Pulmonary embolism (PE) | 415.1 |  |
| Deep vein thrombosis (DVT) | 453.1,453.2, 453.4, 453.5, 453.7-453.9 |  |
| Major bleeding |  |  |
| Intracranial hemorrhage (ICH) | 430, 431, 432 |  |
| Gastrointestinal bleeding (GI bleeding) | 531.0, 531.2, 531.4, 531.6,  532.0, 532.2, 532.4, 532.6,  533.0, 533.2, 533.4, 533.6,  534.0, 534.2, 534.4, 534.6,  535.x1, 537.83, 562.02, 562.03, 562.12 562.13, 568.81, 569.3, 569.85, 578 |  |
| Bleeding of other critical sites | 423.0, 459.0, 593.81, 599.7, 719.1, 784.7, 784.8, 786.3 |  |
| Myocardial infarction | 410, 412 |  |
| Peripheral vascular disease | 441, 443.9, 785.4, V43.4 |  |
| Cerebrovascular disease (CVA) | 430-438 |  |
| Transient ischemic attack (TIA) | 435 |  |
| Dementia | 290, 294.0-294.2, 331 |  |
| Chronic obstructive pulmonary disease (COPD) | 490-496, 500-505, 506.4 |  |
| Connective tissue disease | 710, 714, 725 |  |
| Peptic ulcer disease | 531-534 |  |
| Liver disease |  |  |
| Mild | 571.2, 571.4-571.6 |  |
| Moderate-severe | 456.0-456.2, 572.2-572.8 |  |
| Diabetes mellitus | 250 |  |
| Uncomplicated | 250.0-250.3, 250.7 |  |
| End-organ damage | 250.4-250.6 |  |
| Hemiplegia or paralysis | 342, 344 |  |
| Renal disease | 403-404, 580-586, 588, 590.0, 753.1-753.3, V42.0, V45.1, V56 |  |
| Moderate to severe chronic renal disease (CKD) | 582, 583.0-583.7, 585, 586, 588 |  |
| End stage renal disease | 585.6 |  |
| Acquired immune deficiency syndrome (AIDS) | 042, 079.53 |  |
| Cancer-related treatment |  |  |
| Drug therapy | V58.1, V66.2, V67.2 | 99.25, 99.28 (or including chemotherapy, BRM) |
| Radiotherapy | V58.0, V66.1, V67.1 | 92.2, 92.3 (or including radiotherapy) |
| All Surgery |  | 01-86 (except description mentioned -scopy, care, biopsy, and diagnostic) |
| Inferior Vena Cava (IVC) Filter Placement |  | 38.7 |
| Thrombectomy |  | 39.74 (or including thrombectomy) |
| Central venous catheters |  | 38.97 (or including central venous catheter) |
| Blood transfusions |  | 99.0 |

ICD-9-CM: International Classification of Diseases, Clinical Modification; BRM: Biologic response modifier

# Supplementary Table 11. List of medication codes used in this study

| Description | BNF codes | Medication names |
| --- | --- | --- |
| Anticoagulants | 2.8 |  |
| LMWH |  | Enoxaparin, Nadroparin, Tinzaparin |
| DOACs |  | Apixaban, Dabigatran, Edoxaban, Rivaroxaban |
| Cancer-related drugs | 8.x |  |
| Platinum treatment |  | Carboplatin, Cisplatin, Oxaliplatin |
| Antiplatelet (exclude aspirin) | 2.9 |  |
| NSAIDs (exclude aspirin) | 10.1.1 |  |
| Erythropoietin | 9.1.3 |  |
| Thrombolytics | 2.10 |  |
| EGFR inhibitor |  | Gefitinib, Erlotinib, Afatinib, Brigatinib, Icotinib, Cetuximab, Osimertinib |
| VEGF/VEGF receptor inhibitor |  | Pazopanib, Sunitinib, Bevacizumab, Sorafenib, Regorafenib, Cabozantinib, Ponatinib, Aflibercept, Axitinib, tivozanib, Ramucirumab |
| CYP3A4/P-glycoprotein inducer/inhibitor |  | Amiodarone, Verapamil, Ritonavir, Darunavir, Saquinavir, Itraconazole, Voriconazole, Posaconazole, Ketoconazole, Fluconazole, Erythromycin, Rifampicin, Tacrolimus, Phenytoin, Carbamazepine |
| SSRIs/SNRIs |  | Bupropion, Citalopram, Desvenlafaxine, Duloxetine, Escitalopram, Fluoxetine, Fluvoxamine, Paroxetine, Sertraline, Venlafaxine |

BNF: British National Formulary; LMWH: Low molecular weight heparin; DOACs: Direct Oral Anticoagulants; NSAIDs: Non-steroidal anti-inflammatory drugs; EGFR: Epidermal growth factor receptor; VEGF: Vascular endothelial growth factor; CYP3A4: Cytochrome P450 3A4; P-gp: P-glycoprotein; SSRIs: Selective serotonin reuptake inhibitors; SNRIs: Serotonin and norepinephrine reuptake inhibitors

# Supplementary Table 12. Charlson Comorbidity Index calculation list:

| Factors | Specify | Charlson Comorbidity Index Score |
| --- | --- | --- |
| Age | 50-59 | +1 |
|  | 60-69 | +2 |
|  | 70-79 | +3 |
|  | >=80 | +4 |
| Myocardial infarction | Yes | +1 |
| Congestive heart failure | Yes | +1 |
| Peripheral vascular disease | Yes | +1 |
| Cerebrovascular accident or transient ischemic attack | Yes | +1 |
| Dementia | Yes | +1 |
| Chronic obstructive pulmonary disease | Yes | +1 |
| Connective tissue disease | Yes | +1 |
| Peptic ulcer disease | Yes | +1 |
| Liver disease | Mild | +1 |
|  | Moderate to severe | +3 |
| Diabetes mellitus | Uncomplicated | +1 |
|  | End-organ damage | +2 |
| Hemiplegia | Yes | +2 |
| Moderate to severe chronic renal disease | Yes | +2 |
| Solid tumor | Localized | +2 |
|  | Metastatic | +6 |
| Leukemia | Yes | +2 |
| Lymphoma | Yes | +2 |
| Acquired immune deficiency syndrome | Yes | +6 |
